# Mucociliary and long-term particle clearance in airways of patients with immotile cilia

**DOI:** 10.1186/1465-9921-7-10

**Published:** 2006-01-19

**Authors:** Winfried Möller, Karl Häußinger, Löms Ziegler-Heitbrock, Joachim Heyder

**Affiliations:** 1 Institute for Inhalation Biology and Clinical Research Group 'Inflammatory Lung Diseases', GSF – National Research Centre for Environment and Health, Robert Koch Allee 29, D-82131 Gauting-Munich, Germany; 2 Department for Respiratory Medicine, Asklepios Hospital Munich-Gauting, Robert Koch Allee 2, D-82131 Gauting-Munich, Germany; 3 Department of Infection, Immunity and Inflammation, University of Leicester, Medical Sciences Building, Leicester LE1 9HN, UK

## Abstract

Spherical monodisperse ferromagnetic iron oxide particles of 1.9 μm geometric and 4.2 μm aerodynamic diameter were inhaled by seven patients with primary ciliary dyskinesia (PCD) using the shallow bolus technique, and compared to 13 healthy non-smokers (NS) from a previous study. The bolus penetration front depth was limiting to the phase1 dead space volume. In PCD patients deposition was 58+/-8 % after 8 s breath holding time. Particle retention was measured by the magnetopneumographic method over a period of nine months. Particle clearance from the airways showed a fast and a slow phase. In PCD patients airway clearance was retarded and prolonged, 42+/-12 % followed the fast phase with a mean half time of 16.8+/-8.6 hours. The remaining fraction was cleared slowly with a half time of 121+/-25 days. In healthy NS 49+/-9 % of particles were cleared in the fast phase with a mean half time of 3.0+/-1.6 hours, characteristic of an intact mucociliary clearance. There was no difference in the slow clearance phase between PCD patients and healthy NS. Despite non-functioning cilia the effectiveness of airway clearance in PCD patients is comparable to healthy NS, with a prolonged kinetics of one week, which may primarily reflect the effectiveness of cough clearance. This prolonged airway clearance allows longer residence times of bacteria and viruses in the airways and may be one reason for increased frequency of infections in PCD patients.

## Introduction

Mucociliary clearance (MCC) is an integral part of lung defense mechanisms, enabling efficient clearance of inhaled particles, including microorganisms, from the respiratory tract [1,2]. Airway infections and ciliary dysfunctions can lead to impaired mucus transport [3,4] and can thereby enhance the fraction of retained particles, including microorganisms in the airways. In addition, the defect in ion transport across the airway epithelia of cystic fibrosis (CF) patients [5] is thought to impair MCC [6,7], contributing to chronic infection in these patients.

Primary ciliary dyskinesia (PCD) is a pulmonary disorder manifested by abnormal MCC [8,9], in this case due to immotile cilia that do not beat in a coordinated fashion to propel mucus out of the lung. In the last years it has been shown that PCD is a genetic disease characterized by abnormal ciliary ultrastucture and function (microtubular apparatus), impaired MCC, and chronic lung, sinus and middle ear disease [10]. Situs inversus occurs randomly in approximately 50% of subjects with PCD [11]. Yet, despite deficient MCC patients with PCD appear to fare better clinically (i.e., lower infection rates and decline in lung function) than CF patients [12]. It may be that PCD patients have sufficient clearance from their small airways, as suggested by recent studies of Svartengren and colleagues [13], either by very efficient cough clearance that extends out to the small airways or other, as yet undefined, mechanisms capable of transporting mucus in their airways.

Using gamma scintigraphy, MCC has generally been assessed by measuring the rate of removal over time of radiolabelled particles deposited in the lungs following their inhalation under tidal breathing conditions. The rate of particle clearance from the airways is determined by the integral function of the various components comprising the mucociliary escalator (i.e., frequency and coordination of ciliary beating and rheology of airway secretions). Traditionally it has been assumed that particles depositing on the ciliated airways are rapidly cleared by mucociliary clearance during the first 24 hours following deposition, and that any particles remaining in the lung at 24 hours represents alveolar or "non-ciliated airway" deposition [14-16]. More recent studies suggest that the 24-hour retention of radiolabelled inhaled particles, especially in patients with obstructive pulmonary disease, may reflect in part long-term airway retention [17-21], and this was also included in the recent ICRP model of clearance of particles from the lung [22].

Human studies using the bolus inhalation technique have shown that MCC removes all deposited particles larger than 6 μm from the airways within 24 hours. When smaller particles are inhaled, a certain fraction is retained for longer than 24 hours [17,23]. This fraction increases with decreasing particle size. The mechanisms of this long-term clearance of particles from the airways are unclear. In a recent study we have shown that in healthy non-smokers (NS) the kinetics of long-term retained particles from the airways is very slow, and comparable to that of alveolar clearance of the same type of particles [24]. In this study a magnetic labeling method was applied enabling observation times of up to one year without radioactive burden to the subjects.

The purpose of this study was to evaluate both, the short term mucociliary and the long-term clearance kinetics of inhaled magnetic iron oxide particles from the airways of patients with immotile cilia using the magnetic labeling method (Magnetopneumography, MPG) [25,26], and to compare the results to data on healthy NS, obtained from a previous study [24]. The MPG method has been applied to investigate long-term clearance from the lung periphery over a 1 year period [27], which result in clearance half-times of ≈ 120 days for healthy NS and impairment of clearance due to cigarette smoking and interstitial lung diseases. Studies on patients with immotile cilia may provide new insight into the understanding of clearance studies after shallow bolus inhalation.

Studies of airway clearance require a deposition of the test particles predominantly in the airways. Efforts were made to achieve this requirement by controlled inhalation of particle boli at the end of tidal inhalation [28,29]. The phase1 volume of the anatomical dead space was used as a threshold volume for the bolus penetration depth.

## Methods

### Subjects and pulmonary function testing

Seven patients with immotile cilia syndrome (age 35 +/- 12 years) participated in the study. Anamnestic data were collected using a questionnaire based on ATS – recommendations [30], and all subjects were interviewed by a pulmonary specialist. PCD was confirmed by clinical history and ciliary ultrastructural abnormalities observed by electron microscopic investigation of nasal or bronchial biopsies from each patient [31,32]. Two of the PCD patients had situs inversus totalis and five had clinical and radiological evidence of bronchiectasis. During the first month of clearance measurements none of the PCD patients used oral or inhalative steroids.

The protocol was approved by the Ethical Committee of the Medical School of the Ludwig Maximilian University (Munich, Germany), and informed consent from each subject was obtained. Body plethysmography and spirometry were performed using a Jäger Masterlab (Erich Jäger, Würzburg, Germany). Predicted values of conventional lung function parameters were calculated by normalizing to the reference values proposed by the European Community for Steel and Coal [33]. A lung function test and an MPG measurement of the natural ferromagnetic contamination of the lungs of every subject were obtained before inhalation. MPG measurements were performed 30 min, 3 and 6 hours, 1 and 2 days, 1 week, 1, 3, 6 and 9 months after particle inhalation. Reference data of 13 healthy never-smoking subjects (NS, age 37 +/- 11 years) were taken from a previous study [24].

### Volumetric dead space measurement

A fast mass spectrometer (modified magnetic sector field mass spectrometer; DLT 1100 R, Dennis Leigh Technology, Sandbach UK) was used to measure the physiological dead space [34]. A tracer gas mixture, composed of 0.2% C^18^O_2_, 21% O_2_, and 78.8% N_2 _was applied as a single-breath inhalation. CO_2 _labelled with the stable oxygen isotope ^18^O (C^18^O_2_) was completely taken up in the gas exchanging region of the lung, but not from the airways. Therefore C^18^O_2 _was only expired from the dead space of the lung, and not from the alveolar region. Hence, C^18^O_2 _allows the measurement of the respiratory dead space not only in healthy subjects, but also in patients with COPD and lung emphysema [35,36]. The physiological dead space V_FD _was derived from the C^18^O_2 _expirogram using the method of Fowler [37]. In addition the phase1 dead space volume, V_DP1_, was estimated from the C^18^O_2 _concentration drop to below the 95 % level as a closer threshold volume for the conducting airways.

### Magnetic particle generation, inhalation and Magnetopneumographic detection

The system for magnetic particle generation, inhalation and detection in the lung is described in detail in the previous study [24] and will therefore be repeated here only very shortened. 0.5 – 1 mg of spherical monodisperse ferrimagnetic iron oxide particles (Fe_3_O_4_, 4.2 μm aerodynamic, 1.9 μm geometric diameter, σ_g _< 1.1) were deposited in the lungs by controlled voluntary inhalation of a shallow 100 cm^3 ^aerosol bolus using the respiratory aerosol probe (RAP) [29]. The particles were produced by a Spinning Top Aerosol Generator (STAG) [38]. The mean inhalation and exhalation flow rates were kept at 250 ml/s. The volumetric front depth V_F _of the aerosol bolus was adapted to the individual phase1 dead space volume V_DP1_. At the end of inhalation, an 8 s breath hold was performed in order to enhance the particle deposition. The end-inspiratory volume was 1 L above the functional residual capacity (FRC). The lung expansion of the dead space measurements was 90 % TLC and therefore larger compared to that of the aerosol administration. Therefore the volumetric dead space during aerosol administration requires a 10 % reduction, as can be estimated from the data in Bennett et al. [39]. About 20–30 breath were necessary to deposit 0.5 – 1 mg of magnetite particles in the lung.

Directly after inhalation the particles deposited in the lungs were detected by the Magnetopneumographic (MPG) system [26]. The subjects were positioned on a bed with the lungs directly under the magnetizing coils (magnet). Magnetization was carried out in a short magnetic field pulse. The magnetized particles formed remanent magnetic dipoles, oriented parallel to the magnetizing field and therefore produced a weak remanent magnetic field (rmf) of the lung. The subject was moved under a superconducting loop array where the weak magnetic field of the lungs was detected by a superconducting quantum interference device (SQUID). After correcting for natural ferromagnetic contamination, the rmf detected was shown to be a reliable measure of the amount of particles retained within the lungs [40]. Subjects were studied over an 8 – 9 month post-inhalation period.

### Data analysis

Particles deposited in the lung by the shallow bolus technique showed at least two different mechanisms of clearance. The first fast phase happened within the first days and later proceeded into the slow phase of clearance. The course of the clearance curve was fitted by the sum of two exponential functions according to:



where *B*_*0 *_describes the amount or retained magnetic material directly after inhalation, (*1-A*_*S*_) describes the amount of fast cleared material with the time constant *T*_*F*_, *A*_*S *_describes the amount of slowly cleared material, *T*_*S *_is the time constant of the slowly cleared material. Additionally the amount of retained material after 6 hours, 24 hours, 1 week and 9 month (ret6 h, ret24 h, ret1 w and ret9 m) was analyzed. The significance of differences in the data between PCD patients and healthy NS (reference data obtained from [24]) were analyzed by a two-sided t-test.

## Results

### Data of pulmonary function testing, of anatomic dead space and of particle inhalation

Age and lung function data of the seven PCD patients are shown in Table 1. Five of the seven subjects had lung function data in the normal range of healthy subjects. In two of the seven subjects FEV1 was below the 80 % and FEV1/VCmax was below the 70 % threshold of healthy subjects, respectively, classifying them as obstructive patients (moderate COPD, type IIA) according to GOLD recommendations [41]. Mean VCmax and FEV1 were significantly lower in PCD patients compared NS, while RV%TLC was significantly higher. The dead space measurements gave mean values for V_DF _and V_DP1 _at a lung inflation of 90 % TLC of 290 +/- 54 ml and 173 +/- 36 ml, respectively. Among all subjects V_DP1 _shows a high correlation to the body height (cc = 0.78, p < 0.01). The mean aerosol penetration front depth during bolus inhalation was V_F _= 157 +/- 15 ml. The aerosol bolus was administered at the end of a 1 liter breath from FRC, where the mean lung expansion was 67 +/- 9 %. In order to adapt the lung inflation of the dead space measurements to the aerosol inhalation a reduction of the dead space volumes of about 10–15 % is necessary according to data in [39] and our few measurements. The bolus penetration (front depth) in relation to the Fowler dead space and the phase1 dead space is 59 % of V_DF _and 100 % of V_DP1 _as corrected to 70 % TLC lung expansion. After 8 seconds of breath holding time during aerosol inhalation the mean deposition was D_AW _= 58 +/- 8 % compared to 51 +/- 8 % in healthy NS (from [24]). The deposition without breath hold was below 20 %. The bolus penetration depth V_F _and the phase1 dead space volume, V_DP1 _are correlated (cc = 0.82, p < 0.01).

**Table 1:** Age and lung function data of the seven PCD patients involved in the study

	Age	TLC	FRC	RV	VCmax	Rtot	FEV1	FEV1
Subj.	Years	%pred	%pred	%pred	%pred	kPa*s/l	%pred	%VCmax
#1	27	99	96	100	98	0.18	92	82.1
#2	35	110	124	158	90	0.3	68	65.9
#3	34	101	111	118	96	0.22	85	71.2
#4	42	98	112	124	88	0.31	57	52
#5	23	110	121	126	105	0.18	94	72.3
#6	28	91	84	87	93	0.26	93	79.9
#7	57	95	82	92	100	0.12	106	79.6
								
Mean	35.1	100.6	104.3	115.0	95.7**	0.22	85.0*	71.9
SD	11.5	7.2	17.1	24.5	5.9	0.07	16.9	10.5

(*: p < 0.05; **: p < 0.01 for PCD patients versus reference data from a previous study [24]).

The aerodynamic and the geometric particle size was 4.14 +/- 0.36 μm and 1.87 +/- 0.16 μm in PCD patients, respectively. The particle size distribution obtained by sedimentation cell measurements revealed a geometric standard deviation of σ_g _< 1.1, therefore, the particles can be characterized as monodisperse. The particles were very compact (density ρ = 4.9 g/cm^3^), chemically stable, and resist dissolution in physiological saline, in body fluids, and in the lungs for several month.

### Fast clearance of particles from the airways

**Figure 1:**
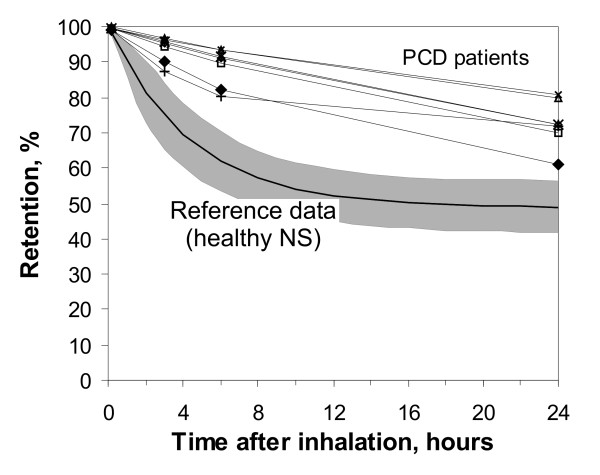
Retention of 2 μm diameter ferromagnetic iron-oxide particles in the airways of seven PCD patients within the first 24 hours (in comparison to reference data from a previous study [24]). Data show individual curves of the PCD patients in comparison to mean values (bold curve) +/- standard deviation (SD) of the reference data.

**Figure 2:**
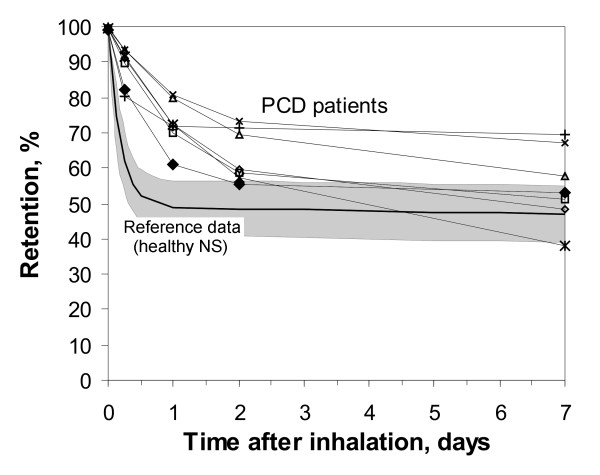
Retention of 2 μm diameter ferromagnetic iron-oxide particles in the airways of seven PCD patients within 1 week post inhalation (in comparison to reference data from a previous study [24]). Data show individual curves of the PCD patients in comparison to mean values +/- standard deviation (SD) of the reference data.

The retention of the ferromagnetic iron oxide particles was measured in the MPG-system directly after, in addition to 3 hours, 6 hours, 1 day, 2 days, 1 week, 1 month, 3 months, 6 months and 9 months after inhalation. The individual retention curves of all seven PCD patients are shown within the first day and within the first week in Figure 1 and in Figure 2, respectively (in comparison to healthy non-smokers from a previous study, mean +/- standard deviation, SD). The data follow a two phase decay with a fast phase within the first week, and a slow phase over the following months. The mean data of the half times of the two phase decay and the fraction of clearance following the slow decay (A_S_) are given in Table 2. After 6 h and after 1 day, 88.8 +/- 5.3 % and 72.6 +/- 6.6 % of the particles were retained in the lung in PCD patients, respectively (64.1 +/- 8.7 % and 49 +/- 8 % in healthy NS [24], p < 0.01). After 1 week, 54.8 +/- 11.0 % of the particles were retained in the lung in PCD patients (45.7 +/- 8.2 % in healthy NS [24], difference not significant, n.s.). Extrapolating the long-term decay back to time zero reveals that 57 +/- 12 % of the particles follow the slow phase of retention (50 +/ 8 % in healthy NS [24], n.s.). Only ≈ 50 % of the particles depend on the mucociliary fast clearance mechanism, which happens with a half time of T_1/2F _= 16.8 +/- 8.6 hours (T_1/2F _= 3.0 +/- 1.6 hours in healthy NS [24], p < 0.01).

**Table 2:** Results of the measurement of the clearance curve parameters

	Unit	Mean +/- SD
*Clearance*		
A_s_		0.57 +/- 0.12
T_1/2f_	hours	16.8 +/- 8.6**
T_1/2s_	days	121 +/- 25
*Retention*		
Ret6 h	%	88.8 +/- 5.3**
Ret1 d	%	72.6 +/- 6.6**
Ret1 w	%	54.8 +/- 11.0
Ret9 m	%	13.9 +/- 5.4

(Ret6 h, 1 d, 1 w and 9 m are retention data after 6 hours, 1 day, 1 week and 9 months; *: p < 0.05; **: p < 0.01 for PCD patients versus reference data from a previous study [24]).

### Slow clearance of particles from the airways

The slow phase of airway clearance of 1.9 μm geometric diameter iron oxide particles is shown in part in Figure 2. Within the first week ≈ 50 % of the particles were cleared via the mucociliary apparatus and the remaining particles followed a mean clearance half time of T_1/2S _= 121 +/- 25 days in PCD patients (T_1/2S _= 109 +/- 78 days in healthy NS, n.s.), 270 days measurement time. After 9 month 14 +/-5.0 % of the initially deposited particles are retained in the lungs of PCD patients (10 +/-9.8 % in healthy NS [24]). Long term airway particle clearance in PCD patients shows no significant difference to healthy NS. Attempts to include an intermediate clearance phase into the model failed.

## Discussion

### Particle deposition

Particle penetration was confined to the phase1 dead space volume of the airways by using the bolus technique. After 8 sec breath holding time deposition was higher in tendency in PCD patients compared to healthy subjects, and correlates with a lower FEV1. As has been shown earlier particle deposition is very sensitive to airway obstructions [42]. Increased particle deposition correlates with higher FEV1 due to narrower airways. This indicates that the PCD patients in this study might have moderate airway obstructions.

### Prolonged airway clearance in PCD patients

Fast mucociliary clearance was finished in healthy NS after 1 day (half time T_1/2F _= 3.0 +/- 1.6 hours), but proceeded in PCD patients for about 1 week (half time T_1/2F _= 16.8 +/- 8.6 hours, p < 0.01), as demonstrated in Figure 1 and 2. After 6 h and after 1 day particles retention is significantly higher in PCD patients showing the inhibition of the clearance mechanism due to non functioning cilia. Our data demonstrate that airway clearance is not completely inhibited in PCD patients, but is slowed and prolonged. In the case of non-functioning cilia, which were confined by ultrastructural investigations, the remaining clearance mechanism may be coughing, as was proposed by other studies [43,44]. Therefore our clearance data in PCD may roughly demonstrate the effectiveness and kinetics of airway clearance and mucus transport by coughing. Compared to clearance by normal mucociliary transport, clearance by coughing, which is the primary clearance mechanism in PCD patients, removed a fraction comparable to that in healthy NS (about 50 % of deposited particles) from the airways, but needs about 1 week in PCD patients, in comparison to less than a day in healthy NS. The frequency of coughing was not monitored in our study, but all PCD patients were coughing all the time. Prolonged airway clearance in PCD patients has also been seen by other authors, but the detection time was limited to 1 day due to the ^99m^Tc labelling method [45], and therefore could not show the full time scale of this process. The impaired and prolonged particle clearance in airways of PCD patients can explain the increased frequency of airway infections, finally resulting in bronchiectasis in many of the patients. As a result of an impaired and prolonged clearance inhaled bacteria and viruses can reside for longer times in the airways, where they can find optimal conditions for growth, due to 37°C body temperature.

In addition we have to keep in mind, that most of the PCD patients were under specific therapies, such as inhalation of saline and of mucolytics, and the application of physiotherapy (positive expiratory pressure breathing, use of flutter device, autogenic drainage breathing) for better detaching mucus from the airways [46]. Therefore the clearance kinetics shown in this study implies the results of these therapies. The clearance may worsen without therapy or during acute airway infections. During the first month of clearance measurements none of the PCD patients used oral steroids, therefore influences on MCC, such as from acute airway infections and drugs can be excluded [47].

### Airway clearance studies in PCD patients in comparison to the bolus technique

Other studies of particles clearance from the airways of PCD patients are controversy. The first studies on patients with immotile cilia report a complete impairment of clearance, while recent studies show up to 80 % particles clearance within 24 hours [4,45]. The differences may be due to the size of the inhaled tracer particles, the method of inhalation and particle deposition in the lung, and the medical treatment. The older studies used single breath inhalation with normal tidal volume, where a large fraction might penetrate down to the lung periphery, where the macrophage mediated long-term clearance mechanism is present. In recent studies particles were deposited by forced inhalation on airway bifurcations with a high central deposition. Particle deposition after inhalation with high flow rates results in faster clearance compared to deposition after slow inhalation and breath holding. In addition nowadays patients are under much better therapies, such as inhalation of saline and of mucolytics, and the application of physiotherapy, resulting in an assistance of cough clearance and a more effective mucus removal from the lung.

Our data show that the aerosol bolus technique gives advantages in understanding the failure of clearance mechanisms involved in PCD by giving a more homogenous distribution of particles within the airways, with a preferred deposition in smaller airways, and by confining the particle deposition primarily to the airways.

### Long term airway clearance

The bronchial clearance measurements after shallow bolus inhalation showing an incomplete airway clearance after 24 hours are still under debate. Despite the possibility that a fraction of particles may reach alveolar structures, even with the use of the shallow bolus technique, a significant fraction of deposited particles in the airways must get lost from the mucociliary escalator. The mechanism underlying the long-term clearance phase can not be completely identified. But, in comparison to other histological studies, we can address airway macrophages as being possible target cells in the long term clearance mechanisms [24,48]. In our previous study on healthy NS we could show that the long-term phase of particle clearance has the same kinetics as clearance of comparable particles from the lung periphery, suggesting for comparable underlying mechanisms. Many of the questions concerning the influence of different ventilation and deposition mechanisms on the long-term airway clearance were discussed in our previous study [24], and will therefore not be repeated here. Studies on patients having immotile cilia can bring further insight into the mechanisms of the retarded clearance.

The detection of long term retained particles in the airways may imply a loss of particles from the mucociliary transport machinery and a transport of deposited particles to the sub-mucus space. Morphometric studies revealed that the particle surface properties and the interaction with surfactant seems to play a key role [49,50]. Deposited particles are coated with surfactant and then get displaced into the aqueous sub-phase, where they may be submerged and penetrate between the cilia. Additionally it was shown that the mucus fluid does not form a continuous layer [51,52]. Particle deposition in such holes allows a direct contact with beating cilia. Such particles can easily be phagocytized by airway macrophages and dendritic cells (DC) [53]. The fact that the fraction of fast cleared particles is not significantly different between PCD patients and healthy NS may suggest that the morphology of mucus and the distribution on the airway surface, including the distribution of patches and holes, may not differ between the PCD patients being involved in our study and healthy subjects. These conditions may change under acute airway infections, resulting in mucus hypersecretion, which is more likely in PCD patients.

As has been shown earlier, the long-term clearance kinetics in the airways coincides with the alveolar clearance kinetics [24], which allows to conclude for macrophage dependent mechanism. Further studies show that a separate population of macrophages can be found in the airways [54,55], which have specific characteristics, and which distinguish them from alveolar macrophages [56,57]. Histological and stereological studies in hamsters have revealed, that already 20 min after inhalation of Latex or Teflon particles, a certain fraction can be found in airway macrophages [58-60], and 24 hours after particle inhalation more than 80 % of the remaining particles are phagocytized by airway macrophages. After an acute aerosol challenge the number of airway macrophages can increase, and therefore enhance the probability of particle uptake by macrophages [60], followed by a long-term retention in airway macrophages and DC. In each subject the long-term clearance was recorded over a 270 days period. There was no statistical difference in the long-term airway clearance kinetics between PCD patients and healthy NS. This may allow concluding that the presence and the function of airway macrophages may not be impaired in the PCD patients being involved in our study. This can change during an acute airway infection, where the number of defence cells (macrophages and neutrophils) can increase.

## Conclusion

Using the shallow bolus technique it has been shown that clearance of particles from the conducting airways shows two distinct phases. Airway particle clearance is prolonged from 1/2 day in healthy NS to 1 week in PCD patients. Mucociliary clearance does not eliminate all particles within the first days after particle deposition, neither in healthy NS nor in PCD patients. Although a certain fraction of the long term retained particles may originate from particle deposition in the lung periphery, the data suggest that part of the long-term clearance mechanism is a function of airway macrophages, and non-functioning cilia do not influence the fraction of long-term retained particles. Since macrophage mediated clearance mechanisms play an important role in the lung periphery, cigarette smoking, lung diseases and drugs which modulate alveolar clearance, may also be of relevance in the airways and have to be investigated in the future.

## Competing interests

The author(s) declare that they have no competing interests.

## Authors' contributions

WM was the principal investigator and performed the studies. KH and LZH performed the clinical part of the study in selecting and classifying the patients. JH contributed to the study design, the evaluation of the data and the preparation of the manuscript. All authors have read the manuscript and accept it in the present form.
